# Molecular characterization of the *SPL* gene family in *Populus trichocarpa*

**DOI:** 10.1186/1471-2229-14-131

**Published:** 2014-05-15

**Authors:** Caili Li, Shanfa Lu

**Affiliations:** 1 Institute of Medicinal Plant Development, Chinese Academy of Medical Sciences & Peking Union Medical College, No.151, Malianwa North Road, Haidian District, Beijing 100193, China

## Abstract

**Background:**

SPLs, a family of transcription factors specific to plants, play vital roles in plant growth and development through regulation of various physiological and biochemical processes. Although *Populus trichocarpa* is a model forest tree, the *PtSPL* gene family has not been systematically studied.

**Results:**

Here we report the identification of 28 full-length *PtSPLs*, which distribute on 14 *P. trichocarpa* chromosomes. Based on the phylogenetic relationships of SPLs in *P. trichocarpa* and *Arabidopsis*, plant SPLs can be classified into 6 groups. Each group contains at least a PtSPL and an AtSPL. The N-terminal zinc finger 1 (Zn1) of SBP domain in group 6 SPLs has four cysteine residues (CCCC-type), while Zn1 of SPLs in the other groups mainly contains three cysteine and one histidine residues (C2HC-type). Comparative analyses of gene structures, conserved motifs and expression patterns of PtSPLs and AtSPLs revealed the conservation of plant SPLs within a group, whereas among groups, the *P. trichocarpa* and *Arabidopsis* SPLs were significantly different. Various conserved motifs were identified in PtSPLs but not found in AtSPLs, suggesting the diversity of plant SPLs. A total of 11 pairs of intrachromosome-duplicated *PtSPLs* were identified, suggesting the importance of gene duplication in *SPL* gene expansion in *P. trichocarpa*. In addition, 18 of the 28 PtSPLs, belonging to G1, G2 and G5, were found to be targets of miR156. Consistently, all of the AtSPLs in these groups are regulated by miR156. It suggests the conservation of miR156-mediated posttranscriptional regulation in plants.

**Conclusions:**

A total of 28 full-length SPLs were identified from the whole genome sequence of *P. trichocarpa*. Through comprehensive analyses of gene structures, phylogenetic relationships, chromosomal locations, conserved motifs, expression patterns and miR156-mediated posttranscriptional regulation, the *PtSPL* gene family was characterized. Our results provide useful information for evolution and biological function of plant SPLs.

## Background

SPL proteins constitute a diverse family of transcription factors playing vital roles in plant growth and development. SPLs are specific to plants and have a highly conserved SBP (SQUAMOSA PROMOTER BINDING PROTEIN) domain with approximately 78 amino acid residues. The domain contains three functionally important motifs, including zinc finger 1 (Zn1), zinc finger 2 (Zn2), and nuclear location signal (NLS) [1,2]. Genes encoding SPLs were first identified for SBP1 and SBP2 in *Antirrhinum majus*[3]. Lately, it has been found in various green plants, including single-celled green algae, mosses, gymnosperms, and angiosperms. The results showed that *SPLs* existed as a large gene family in plants. For instance, the *SPL* gene family in *Arabidopsis*, rice, *Physcomitrella patens*, maize and tomato includes 16, 19, 13, 31 and 15 members, respectively [4-9].

The 16 *Arabidopsis SPLs* are termed as *AtSPL1* to *AtSPL16*[2], respectively, of which *AtSPL1*, *AtSPL7*, *AtSPL12*, *AtSPL14* and *AtSPL16* are relatively large and expressed constitutively, while the others are relatively small and highly expressed in flowers [4,10]. Ten of the 16 *AtSPLs*, including *AtSPL2*–*AtSPL6*, *AtSPL9*–*AtSPL11*, *AtSPL13* and *AtSPL15*, are regulated by miRNAs belonging to the *MIR156* family [11-17]. *AtSPL3*, *AtSPL4* and *AtSPL5* contain complementary sequences of miR156 in 3’ UTR, and all of them promote vegetative phase change and flowering [10,14,18]. *AtSPL2*, *AtSPL10* and *AtSPL11* regulate morphological traits of cauline leaves and flowers [19]. Overexpression of miR156b reduces the accumulation of *AtSPL2*, *AtSPL10* and *AtSPL11* mRNA [12,14,20]. *AtSPL9* and *AtSPL15* act redundantly in controlling the juvenile-to-adult growth phase transition and leaf initiation rate in *Arabidopsis*[21]. Six *AtSPLs*, including *AtSPL1*, *AtSPL7*, *AtSPL8*, *AtSPL12*, *AtSPL14* and *AtSPL16*, are not targets of miR156 in *Arabidopsis*. Among them, *AtSPL7* can bind directly to the Cu-response element (CuRE) containing a core sequence of GTAC and is a regulator of Cu homeostasis in *Arabidopsis*[22]. *AtSPL8* regulates pollen sac development [23], male fertility [24], GA biosynthesis and signaling [25]. *AtSPL14* plays significant roles in plant development and sensitivity to fumonisin B1 [26]. Among the 19 rice *SPLs*, half are predominantly expressed in various young organs [27]. OsSPLs targeted by miR156 are involved in the development of flowers in rice. *OsSPL14* regulated by miR156 also controls shoot branching in the vegetative stage [8,28,29]. In maize, *liguleless1*containing the SBP domain regulates ligule and auricle formation [30,31].

*Populus trichocarpa* is a model plant with whole genome sequence available [32]. A total of 352 miRNA precursors, including 12 for miR156, have been identified [33-39]. However, the regulation of miR156 in *P. trichocarpa PtSPLs* has not been analyzed. In our previous studies [40], 17 PtSPLs, which appeared to be full-length or partial sequence with at least 300 amino acids, were identified from the *Populus* genome assembly v1.1 (http://genome.jgi-psf.org/Poptr1_1/Poptr1_1.home.html). They were named PtSPL1–PtSPL17, respectively, of which PtSPL3 and PtSPL4 had the highest similarities with AtSPL7 involved in Cu homeostasis [40]. In order to characterize the whole *SPL* gene family in *P. trichocarpa*, we searched the *Populus* genome assembly v1.1, v2.2 and v3.0 [32]. It resulted in the identification of 28 full-length *PtSPLs*. Gene structures, chromosomal locations, phylogenetic relationships, conserved protein motifs and expression patterns of all identified *PtSPLs* were systematically analyzed. MiR156-mediated posttranscriptional regulation of *PtSPL* genes was investigated. The results provide useful information for elucidating the biological functions of *SPLs* in *P. trichocarpa*.

## Results

### Identification of 28 *SPL* genes in *P. trichocarpa* genome

Analysis of the *Populus* genome assembly v1.1, v2.2 and v3.0 showed the existence of 28 full-length *SPL* genes in the *P. trichocarpa* genome (Table 1). All of the deduced PtSPL proteins contained the conserved SBP domain. The theoretical p*I* of deduced PtSPL proteins ranged from 5.87 to 9.49. The length varied between 148 and 1044 amino acids. The molecular weight (Mw) varied from 16.2 to 116.1 kDa (Additional file 1). The distribution of p*I* is similar to AtSPLs (Additional file 2); however, the length and Mw of PtSPLs are larger than AtSPLs.

**Table 1:** ***PtSPL *** **gene names and gene model IDs in the *Populus *** **genome assembly v1.1, v2.2 and v3.0**

**Gene name**	**Gene ID**		
	**V1.1**	**V2.2**	**V3.0**
*PtSPL1*	GW1.X.791.1	POPTR_0010s16370	Potri.010G154000
*PtSPL2*	FGENESH4_PM.C_LG_II000008	POPTR_0002s00440	Potri.002G002400
*PtSPL3*	ESTEXT_FGENESH4_PM.C_LG_X0096	POPTR_0010s02710	Potri.010G026200
*PtSPL4*	ESTEXT_FGENESH4_PM.C_LG_VIII0830	POPTR_0008s20160	Potri.008G197000
*PtSPL5*^a^	GRAIL3.0010027501 ^b^ + GRAIL3.0010027301^b^ + GRAIL3.0010027401^b^	POPTR_0008s09810	Potri.008G098600
*PtSPL6*^a^	ESTEXT_GENEWISE1_V1.C_LG_XIV2145^b^ + GW1.XIV.2149.1^b^	POPTR_0014s10960	Potri.014G114300
*PtSPL7*^a^	GRAIL3.0050015101^b^ + GW1.8978.5.1^b^ + GW1.II.489.1^b^	POPTR_0002s18970	Potri.002G188700
*PtSPL8*	FGENESH4_PG.C_LG_II001303	POPTR_0002s14330	Potri.002G142400
*PtSPL9*^a^	EUGENE3.00051637^b^ + EUGENE3.00051638^b^	POPTR_0005s28010	Potri.005G258700
*PtSPL11*	GRAIL3.0047015901^b^	POPTR_0003s17120	Potri.003G172600^b^
*PtSPL12*	GRAIL3.0010026901	POPTR_0008s09750	Potri.008G097900
*PtSPL13*	FGENESH4_PG.C_LG_X001404	POPTR_0010s16400	Potri.010G154300
*PtSPL14*	ESTEXT_GENEWISE1_V1.C_LG_XV2187	POPTR_0015s11100	Potri.015G098900
*PtSPL15*	EUGENE3.00120942	POPTR_0012s10260	Potri.012G100700
*PtSPL16*	ESTEXT_GENEWISE1_V1.C_1240186	POPTR_0011s05480	Potri.011G055900
*PtSPL17*^a^	EUGENE3.00160416	POPTR_0016s04880^b^ + POPTR_0016s04890^b^	Potri.016G048500^c^
*PtSPL18*	GW1.I.7783.1^b^	POPTR_0001s13630	Potri.001G058600
*PtSPL19*	GW1.I.7690.1^b^	POPTR_0001s13890	Potri.001G055900
*PtSPL20*	GW1.107.39.1^b^	POPTR_0001s40870	Potri.001G398200
*PtSPL21*	GW1.II.3778.1^b^	POPTR_0002s14320	Potri.002G142200
*PtSPL22*	GW1.III.2396.1^b^	POPTR_0003s16780	Potri.003G169400
*PtSPL23*	GW1.IV.3037.1^b^	POPTR_0004s04630	Potri.004G046700
*PtSPL24*	GW1.VII.548.1^b^	POPTR_0007s01030	Potri.007G138800
*PtSPL25*	GW1.XI.3794.1^b^	POPTR_0011s11770	Potri.011G116800^d^
*PtSPL26*	GW1.40.81.1^b^	POPTR_0014s05680	Potri.014G057700
*PtSPL27*	GW1.40.76.1^b^	POPTR_0014s05690	Potri.014G057800
*PtSPL28*	GW1.129.152.1^b^	POPTR_0015s07140	Potri.015G060400
*PtSPL29*	GW1.164.76.1^b^	POPTR_0018s14680	Potri.018G149900

^a^Genes are split into 2 or 3 gene models in v1.1 or v2.2;

^b^Gene models with partial sequence;

^c^The gene model includes 7 additional amino acids at the N-terminal compared with the model in v1.1.

^d^The gene model includes 49 additional amino acids at the N-terminal compared with the model in v2.2.

**Figure 1:**
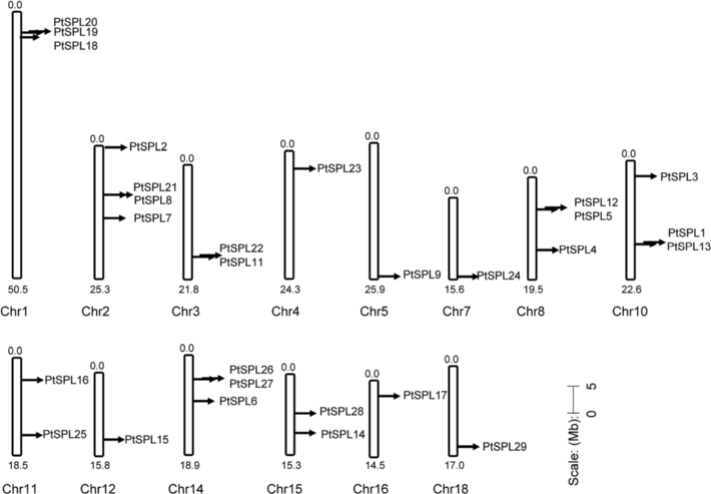
**Chromosomal location of *PtSPL *genes.** Scale represents a 5 Mb chromosomal distance.

Mapping *PtSPLs* to the *P. trhichocarpa* genome showed that 28 *PtSPLs* were unevenly distributed on 14 chromosomes with four on Chr2, 3 on each of Chr1, Chr8, Chr10 and Chr14, 2 on each of Chr3, Chr11 and Chr15, and one on each of Chr4, Chr5, Chr7, Chr12, Chr16 and Chr18 (Figure 1). Relatively high densities of *PtSPLs* were observed in the top and bottom regions of Chr8, Chr10, Chr11 and Chr14, the top of Chr1, Chr4, and Chr16, and the bottom of Chr3, Chr5, Chr7, Chr12 and Chr18. Few are in the central regions of chromosomes. Moreover, 11 pair of *PtSPLs* (Ks < 1.0) were evolved from intrachromosomal duplication (Table 2), indicating the importance of gene duplication for *PtSPL* gene expansion.

**Table 2:** **Estimated age of the duplication events for *PtSPL *** **paralogous genes**

**Paralogous genes**	**Ks**	**Estimated time (mya)**
*PtSPL19*(Chr1)/*PtSPL11*(Chr3)	0.16	9
*PtSPL21*(Chr2)/*PtSPL26*(Chr14)	0.31	17
*PtSPL8*(Chr2)/*PtSPL27*(Chr14)	0.29	16
*PtSPL2*(Chr2)/*PtSPL9*(Chr5)	0.27	15
*PtSPL12*(Chr8)/*PtSPL13*(Chr10)	0.24	13
*PtSPL5*(Chr8) /*PtSPL1*(Chr10)	0.22	12
*PtSPL18*(Chr1)/*PtSPL22*(Chr3)	0.17	9
*PtSPL23*(Chr 4)/*PtSPL16*(Chr11)	0.22	12
*PtSPL20*(Chr1)/*PtSPL25*(Chr11)	0.39	21
*PtSPL7* (Chr2)/ *PtSPL6* (Chr14)	0.25	13
*PtSPL15*(Chr12)/*PtSPL14*(Chr15)	0.30	16

### Phylogenetic analysis of SPLs in *P. trichocarpa* and *Arabidopsis*

**Figure 2:**
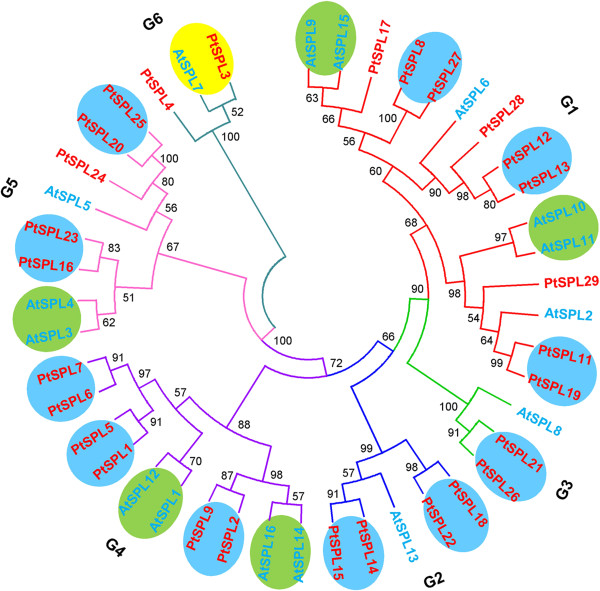
**Neighbor-joining (NJ) phylogenetic tree for 44 SPLs in *P. trichocarpa *and *Arabidopsis*.** The groups of homologous genes identified and bootstrap values are shown. The reliability of branching was assessed by the bootstrap re-sampling method using 1,000 bootstrap replicates. Bootstrap values are shown below nodes.

**Figure 3:**
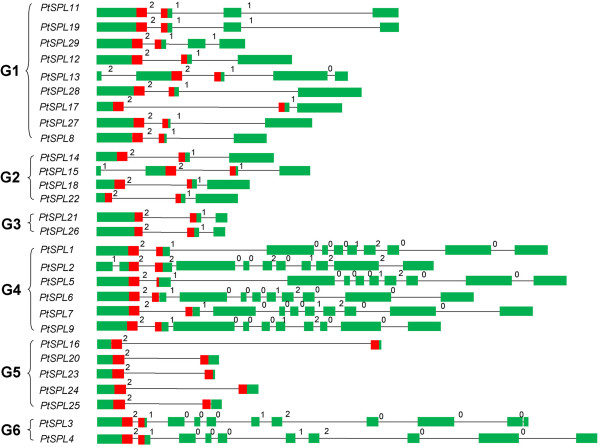
**Exon-intron structures of *PtSPLs*.** Introns are represented by lines. Exons are indicated by green boxes. The SBP domains are shown in red boxes. Intron phases are shown by 0, 1 and 2.

In order to investigate the evolutionary relationship between *P. trichocarpa* and *A. thaliana* SPL proteins, a neighbor-joining (NJ) phylogenetic tree was constructed for 28 PtSPLs and 16 AtSPLs using MEGA5.1. The reliability of branching was assessed by the bootstrap re-sampling method using 1,000 bootstrap replicates. Only nodes supported by bootstrap values >50% are used for further analysis. The results showed that the 44 SPL proteins clustered into 6 groups (named G1–G6), each of which contained at least one AtSPL and one PtSPL (Figure 2). It is consistent with the results from *SmSPLs* in *Salvia miltiorrhiza*[41]. To further confirm that there are 6 groups of SPLs, we also constructed a phylogenetic tree for 28 PtSPLs, 16 AtSPLs, 18 rice OsSPLs and 15 SmSPLs. As shown in Additional file 3, the 77 SPLs also clustered into 6 groups. The difference between the two trees constructed (Figure 2, Additional file 3) is that PtSPL12, PtSPL13, PtSPL28 and AtSPL6 belonging to G1 in Figure 2 are included in G2 in Additional file 3. An intron was found in the SBP domain-encoding region of all *SPL* genes from *P. trichocarpa* and *Arabidopsis* (Figure 3); however, sequence feature analysis showed that the SBP domain of SPLs in G6 (AtSPL7, PtSPL3 and PtSPL4) were divergent from the other groups. The N-terminal zinc finger of G6 SPLs has four cysteine residues in the SBP domain, while SPLs in the other groups mainly contain three cysteines and one histidine, indicating the diversification of plant SPL evolution. On the other hand, *SPLs* within a group have similar intron number, exon-intron structure, and coding sequence length. Consistently, the length, Mw and theoretical p*I* of deduced SPL proteins within a group are also similar, although they are divergent among groups. It suggests the conservation of plant SPLs in a group. Phylogenetic analysis showed that PtSPL3 and PtSPL4 had high homology with AtSPL7, an *Arabidopsis* SPL with the capability of binding CuREs in the *MIR398* promoter *in vitro* and involved in response to copper deficiency in *Arabidopsis*[22]. It is consistent with our previous results for PtSPLs [40]. Based on the phylogenetic tree, PtSPL3 and AtSPL7 are very likely to be orthologous proteins (Figure 2). Additionally, 5 pairs of AtSPLs and 11 pairs of PtSPLs seem to be paralogous proteins (Figure 2). It includes AtSPL9/15, AtSPL10/11, PtSPL8/27, PtSPL12/13 and PtSPL11/19 belonging to G1, PtSPL18/22 and PtSPL14/15 from G2, PtSPL21/26 belonging to G3, AtSPL14/16, AtSPL1/12, PtSPL2/9, PtSPL1/5 and PtSPL6/7 included in G4, and AtSPL3/4, PtSPL16/23 and PtSPL20/25 clustering in G5. About 62.5% of the 16 AtSPLs and 78.5% of the 28 PtSPLs exist as paralogous pairs. It suggested that the expansion of *SPL* genes occurred after separation of paralogous genes. The results from paralogous pair identification were consistent with segmental duplications in the *P. trichocarpa* genome (http://chibba.agtec.uga.edu/duplication/) [32], suggesting the origination of paralogous *PtSPLs* from segmental duplication. Prediction of potential age of tandem duplication events using synonymous substitutions (Ks) values showed that the segmental duplication events for *PtSPLs* appeared to occur in 9–21 mya (Table 2). It is consistent with the age of *P. trichocarpa* genome duplication events [32].

### Comparative analysis of *PtSPL* and *AtSPL* gene structures

Gene structure analysis showed that the number of introns in the coding region of 28 *PtSPL* genes varied from 1 to 10. The number of *PtSPLs* with 1, 2, 3, 4, 9 and 10 introns is five, ten, four, one, six, and two, respectively (Figure 3, Additional file 1). Similarly, the intron number of 16 *AtSPLs* varies between 1 and 9 (Additional file 2). The pattern of intron distribution in *PtSPLs* is quite similar to *AtSPLs* with the majority to be 2 and 9 introns, followed by 1 and 3 (Figure 3, Additional files 1 and 2) [41]. In addition, the position of intron in the SBP domain is highly conserved. It locates in the codon for the 48th amino acid of SBP domain (Additional file 4). These results suggest the conservation of exon-intron structures between *PtSPLs* and *AtSPLs*.

**Figure 4:**
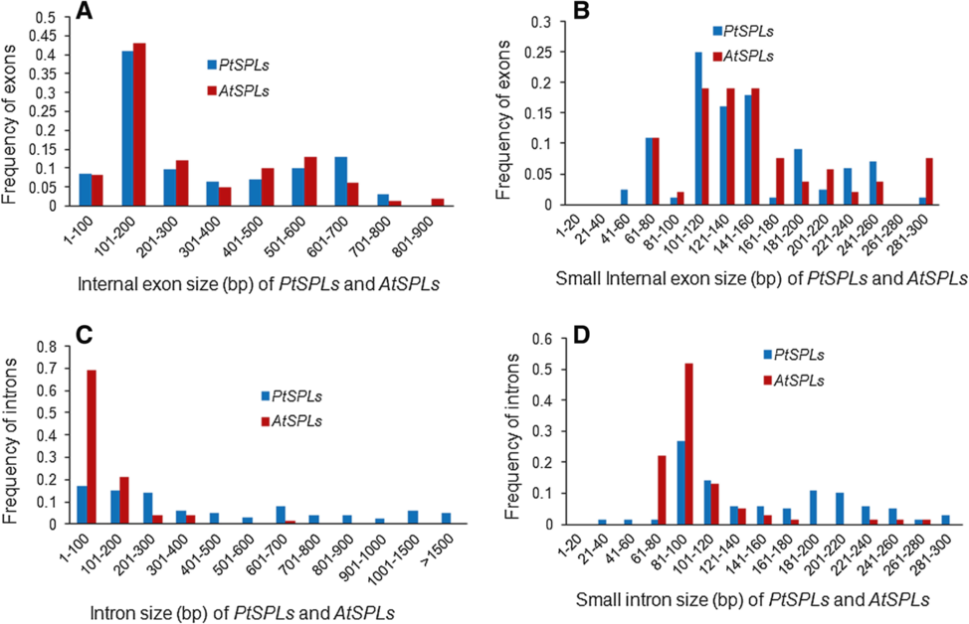
**Size distribution of exons and introns in *PtSPLs *and *AtSPLs*. A**: Size distribution of exons in *PtSPLs* and *AtSPLs*; **B**: Detailed size distribution of small exons in *PtSPLs* and *AtSPLs*; **C**: Size distribution of introns in *PtSPLs* and *AtSPLs*; and **D**: Detailed size distribution of small introns in *PtSPLs* and *AtSPLs*.

The length of introns varies significantly among *PtSPL* genes, such as those in G1, G2 and G5 (Figure 3). We analyzed the internal exons and introns of *PtSPLs* and *AtSPLs*. The results showed that the exons of *PtSPLs* had a size from 43 to 884 bp with an average of 314 bp, which is slightly greater than 293 bp of the average length of *AtSPL* exons. Approximately 59% of *PtSPL* exons and 63% of *AtSPL* exons have a size below 300 bp and 71% and 70% of exons are between 60 and 160 bp in *PtSPLs* and *AtSPLs*, respectively (Figure 4). Although the size distribution of *PtSPLs* exons is similarity with *AtSPL* exons, intron size distribution is more variable, ranging from 30 bp to 3.0 kb. There are 6 *PtSPL* introns (5%) with sizes >1.5 kb; however, no such introns exist in *AtSPLs*. About 55% of *PtSPLs* have sizes below 300 bp and 56% of introns are between 60 and 160 bp; however, the majority of *AtSPLs* (94%) have sizes below 300 bp. The average size of *PtSPL* introns is 476 bp, which is much greater than 120 bp of *AtSPLs*. These results suggest the difference of exon and intron size distribution between *PtSPLs* and *AtSPLs*.

### Identification of 25 conserved motifs

**Figure 5:**
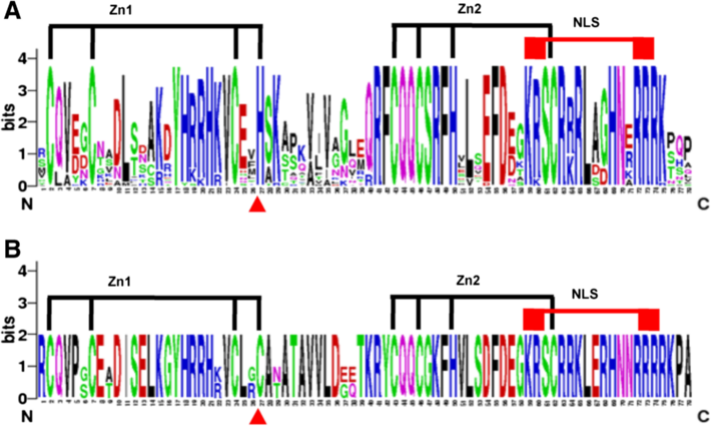
**Sequence logo of the SBP domain of PtSPLs. A**: Sequence logo of the SBP domain of PtSPLs in G1–G5; **B**: Sequence logo of the SBP domain of PtSPLs in G6. Two conserved Zn-finger structures and the NLS are indicated.

Conserved domains of PtSPLs were analyzed using Pfam (http://pfam.sanger.ac.uk) and by BLAST analysis of protein sequences against the Conserved Domain Database (CDD, http://www.ncbi.nlm.nih.gov/Structure/cdd/wrpsb.cgi). The results showed that all of the 28 PtSPLs and 16 AtSPLs contained a SBP domain with about 78 amino acid residues in length (Figure 5). It is not surprising given that the SBP domain was used for *PtSPL* identification. Sequence analysis of SBP domains revealed that the conserved zinc-binding sites, Zn1 and Zn2, also existed in the SBP domain of PtSPLs (Figure 5). Zn1 is Cys3His-type (CCCH-type) in G1–G5 SPLs (Figure 5A); however, the His residue in Zn1 is replaced by a Cys residue in G6, which results in the signature sequence of G6 SPLs to be CCCC (Figure 5B). Unlike Zn1, the signature sequence (C2HC) of Zn2 is highly conserved in all SPLs analyzed. In addition to Zn1 and Zn2, the SBP domain contains a conserved nuclear location signal (NLS) in the C-terminus of SBP domains (Figure 5). The conservation of SBP domains between PtSPLs and AtSPLs indicates that the domain organization has been established in ancient plants. Moreover, six PtSPLs (PtSPL1, PtSPL2, PtSPL5, PtSPL6, PtSPL7 and PtSPL9) belonging to G4 contain an ANK or Ank-2 domain with three or four ankyrin repeats (Additional file 5), which are involved in protein-protein interaction [42]. It is consistent with previous results from AtSPLs and SmSPLs [41].

**Figure 6:**
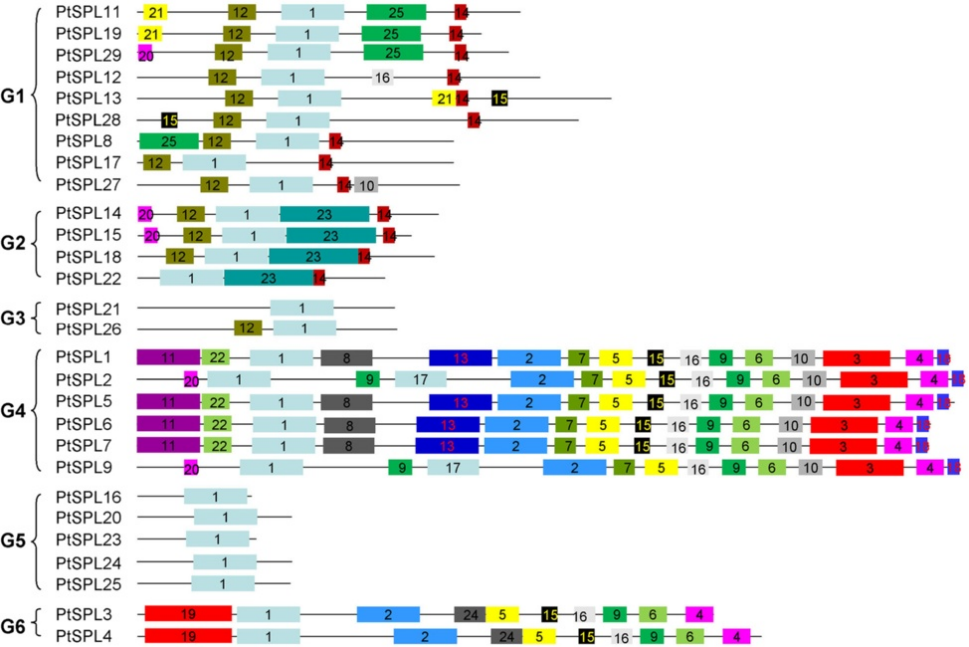
**Distribution of conserved motifs in PtSPLs.** Motifs represented with boxes are predicted using MEME. The number in boxes (1–25) represents motif 1–motif 25, respectively. Box size indicates the length of motifs.

In addition to the conserved domains, other conserved motifs could also be important for the function of SPLs [27,43]. We searched conserved motifs using MEME and applied an e-value cut off of 1e^−10^ to the recognition. It resulted in the identification of 25 motifs for 28 PtSPLs (Figure 6, Table 3). The majority of motifs identified are conserved between PtSPLs and AtSPLs [41], while three, including motifs 11, 19 and 23, are specific to PtSPLs. It indicates the conservation and diversity of PtSPLs and AtSPLs. The number of motifs in each SPL varies from 1 to 16 (Figure 6). Motif 1 is actually the SBP domain. Consistently, it exists in all SPLs analyzed. Motif 14 existed in G1 and G2 SPLs contains the target gene sequence of miR156, indicating the posttranscriptional regulation of G1 and G2 SPLs by miR156. In addition to motifs 1 and 14, several motifs widely exist in two SPL groups, such as motif 12 found in G1 and G2, motifs 2, 4, 5, 6, 15 and 16 existing in G4 and G6 (Figure 6), indicating the importance of these motifs. We also found several motifs to be group-unique, such as motif 24 specifically existing in G6 SPLs and motifs 7, 9, 10 and 18 specific to G4 (Figure 6). These group-unique motifs could be important for specific roles of SPLs in the group. Moreover, PtSPLs and AtSPLs [41] within a group share similar motif(s), indicating they probably play similar roles in plant growth and development.

**Table 3:** E-value and consensus sequences of 25 motifs identified in PtSPLs

**Motif**	**E-value**	**Consensus sequence**
1	1.0e-2549	CQVEGCNADLSSAKDYHRRHKVCEVHSKAPKVIVAGLEQRFCQQCSRFHLLSEFDEGKRSCRRRLAGHNERRRKPQPD
2	3.3e-516	SDQPSSSSSSGDAQCRTGRIVFKLFDKDPNDFPGTLRTQILDWLSHSTDMESYIRPGCIILTIYLAMPEAAWEELCCDLG
3	6.2e-387	LFRPDVAGPAGLTPLHIAACKDGSEDVLDALTEDPGEVGISAWKNARDATGFTPYARLRGHHSYIHLVQRKLADKRNGQVSVVI
4	3.6e-174	ASRSLLYRPAMLSMVAIAAVCVALLFKSCPEVLYV
5	9.0e-136	VEAGEETEFVVKGRNLYQPGTRLLCAVEGKYLVQETTQALMD
6	7.4e-135	FPLRRFKFLLEFSMDRDWCAVVRKLLDMLVEGNVCRD
7	5.6e-100	FWRTGWFYVRVQNQLAFHKNGQVVLDTSL
8	3.0e-085	GGSMNDDQGYLLTSILSNLHSNRSDQTKDQDLLSHLLRSLASHAGEHNGRNLFGLLQGPRGL
9	1.8e-095	EGMPSKEQALDFLNEIGWLLHRSDLKSRL
10	4.8e-088	SSLEALSEMGLLHRAVRRNSRKMVELLLR
11	7.4e-085	MEARFGGESHHFYAPVPSDLKAVGKRGLEWDLNDWKWDGDLFIASPLNPVPSDCRSRQFFPTGPGLGEKAGGNNSNSSCS
12	3.2e-077	STSLGASxSSGESLLGLKLGKRIYFEDAxGxNNxK
13	3.5e-070	FSIPNNFAAKSEEPEATAGQIKLNFDLNDIYDDSDDGIEDIERSHAPVNAGMGSFDCPLMVQQDSHKSSPPHTSGNSDS
14	1.3e-052	ASDSDCALSLLSSQS
15	8.9e-057	NFSCSxPNLLGRGFIEVED
16	9.8e-064	PFIIADADVCSEIRILEQEFD
17	8.7e-053	GERISSCNESPSEDSDSQGQDSRPNLPLQLFSSSPENESRPKVASSRKYFSSASSNPIEDRSPSSSP
18	1.8e-039	PFRWELLDYGT
19	5.1e-035	QHDGDMEIHLPPITTDWDWGDILDFAVDDQFPLSFDTPGDLTQPIDNPTPEIESQQLEAPVPDRVRKRDPRLTCSNFLAGIVPCACPEMDELLLEEEAALPGKKRVRVARAG
20	2.8e-033	DDWNLKAWDWDGDEFEA
21	8.5e-032	MDCNGKPHLQWDWENLIMFNAITTENSKK
22	2.7e-030	DEDNLGDEKGKRELEKRRRVVFIDDDNLND
23	3.3e-028	VNSARIFSNQGTRYLHFGSSQIFSTSAMNAAWTGAAKAERDPMLNTSQSSMNFDGRKNLFPGSLSPNYKEGKQFPFLQGTSSTIPGDSIHLDANSTLGNSQKMFSDGLNR
24	3.4e-027	KGRMRVYLNNMIFNVTKDGHSVMKVNVKGHAPRLHYVHPTC
25	5.9e-025	DERQQMSHAWDKAPLVHARPNANLTWEGTSISKFTITKDYIAKPAEIGGNDGQFHLPGFDLTNGIATQHHHKSN

### Expression patterns of *SPLs* in *P. trichocarpa*

**Figure 7:**
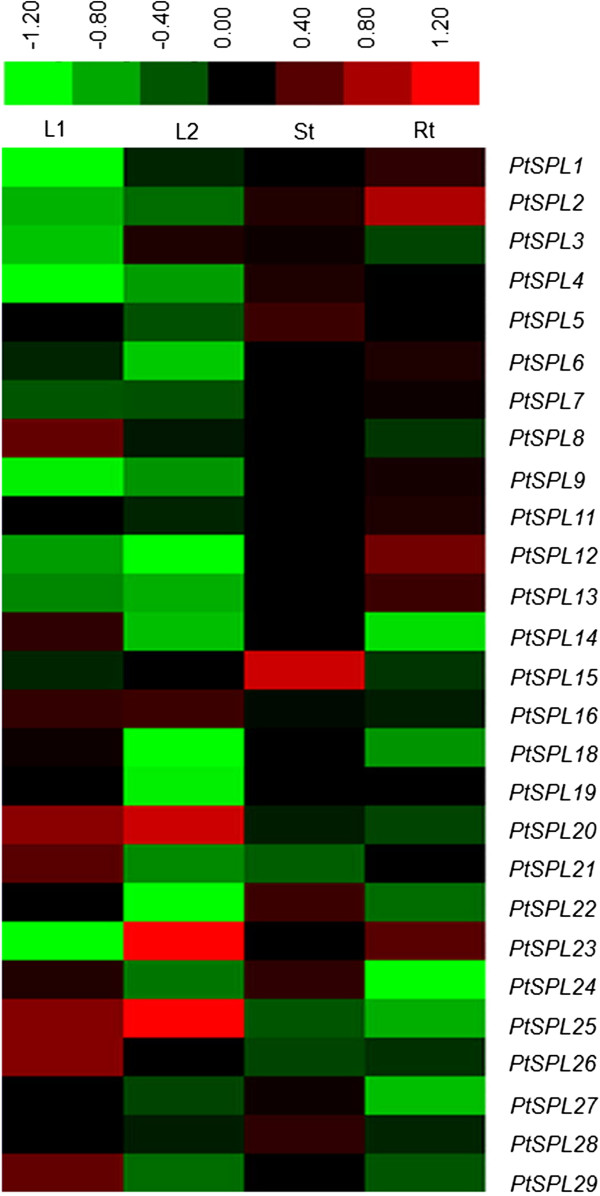
**Expression patterns of *PtSPLs *in four tissues of *Populus trichocarpa*.** Microarray data was obtained from PopGenIE [44] and analyzed using the average linkage clustering technique in Cluster 3.0 [75]. Color scale represents log2 expression values. Green indicates that the expression levels of *PtSPLs* are low; while red indicates that the levels are high. Rt, roots; St, young stems; L1, young leaves; L2, mature leaves.

**Figure 8:**
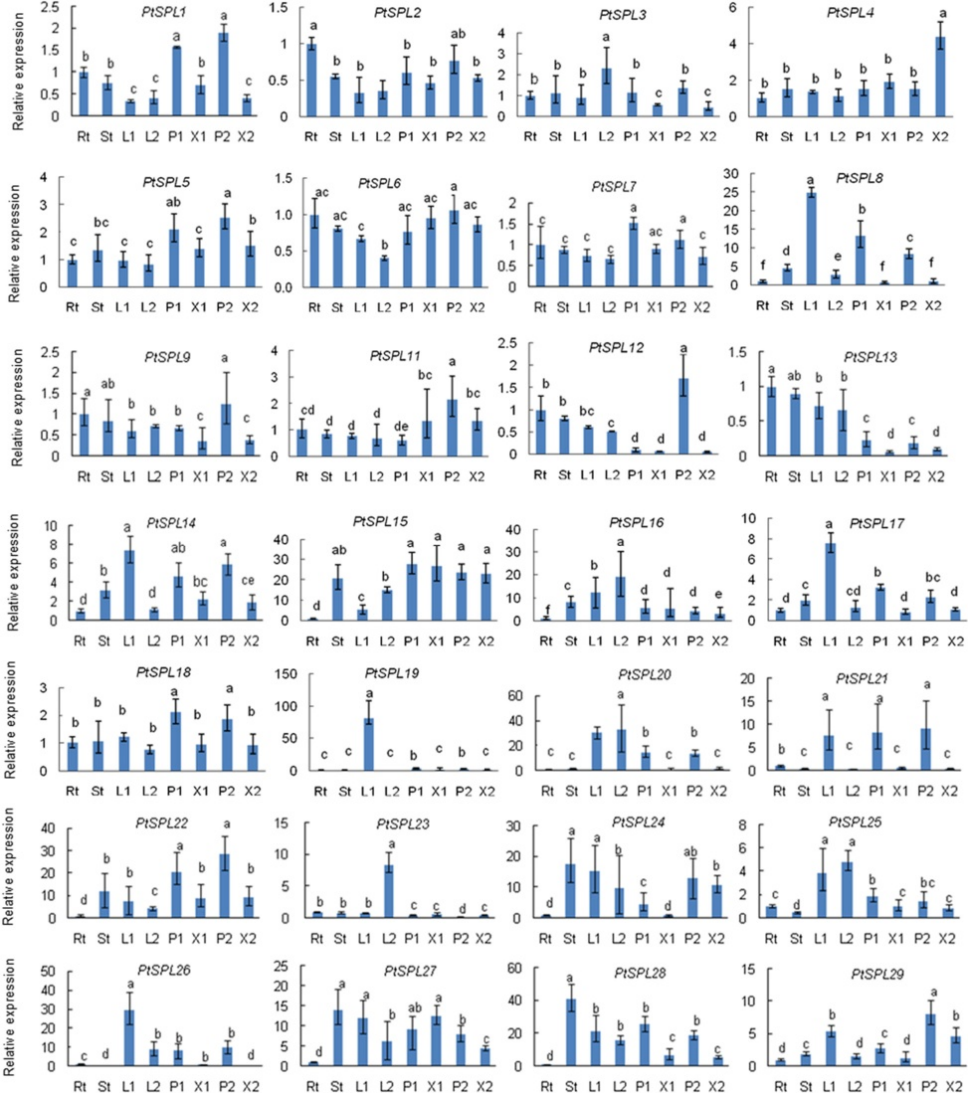
**Analysis of *PtSPL *gene expression in eight tissues of *Populus trhichocarpa *using the qRT-PCR method.** Fold changes of transcript levels in young root (Rt), young stems (St), young leaves (L1), mature leaves (L2), developing secondary xylem (X1), developing secondary phloem (P1), developed secondary xylem (X2) and developed secondary phloem (P2) of *Populus* plants are shown. Transcript levels in roots were arbitrarily set to 1 and the levels in other tissues were given relative to this. Error bars represent standard deviations of mean value from three biological replicates. ANOVA (analysis of variance) was calculated using SPSS. P < 0.05 was considered statistically significant.

The expression pattern of a gene is often correlated with its function. In order to preliminarily elucidate the roles of *PtSPLs* in *P. trichocarpa* development, we first searched PopGenIE for gene expression data from microarray analysis [44]. Except for *PtSPL17*, the expression levels of 27 *PtSPLs* in roots, stems, young leaves and mature leaves were obtained (Figure 7). Next, we examined the relative expression levels of 28 *PtSPLs* in young leaves, mature leaves, young stems, young roots and tissues from developing secondary xylem and phloem from the 4th–6th and 12th–25th internodes of one-year-old *P. trichocarpa* plants using the quantitative real-time RT-PCR method (Figure 8). The results showed that qRT-PCR data was generally consistent with microarray data for relative expression of *PtSPLs* in roots, stems, young leaves and mature leaves (Figures 7 and 8). Although all *PtSPLs* were expressed in at least one of the tissues examined, differential expression was observed. Many putative paralogous genes, such as *PtSPL18/22* in G2, *PtSPL21/26* in G3, *PtSPL2/9*, *PtSPL1/5* and *PtSPL6/7* in G4 and *PtSPL16/23* belonging to G5, show similar expression patterns, suggesting redundant roles of these *PtSPL* gene pairs. However, the expression patterns of few gene pairs, including *PtSPL12/13* in G1, and *PtSPL14/15* belonging to G2 are distinct. It indicates these *PtSPLs* may play different roles in *P. trichocarpa* development, although they are paralogous genes.

### MiR156-mediated posttranscriptional regulation of *PtSPLs*

**Figure 9:**
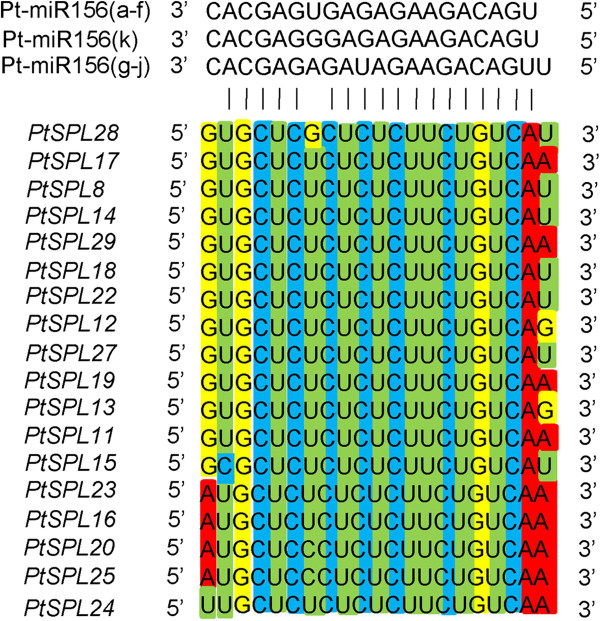
**Sequence alignment of *P. trichocarpa *** **miR156a–miR156j with their complementary sequence in coding regions and 3’ UTRs of 18 *PtSPLs*.**

**Figure 10:**
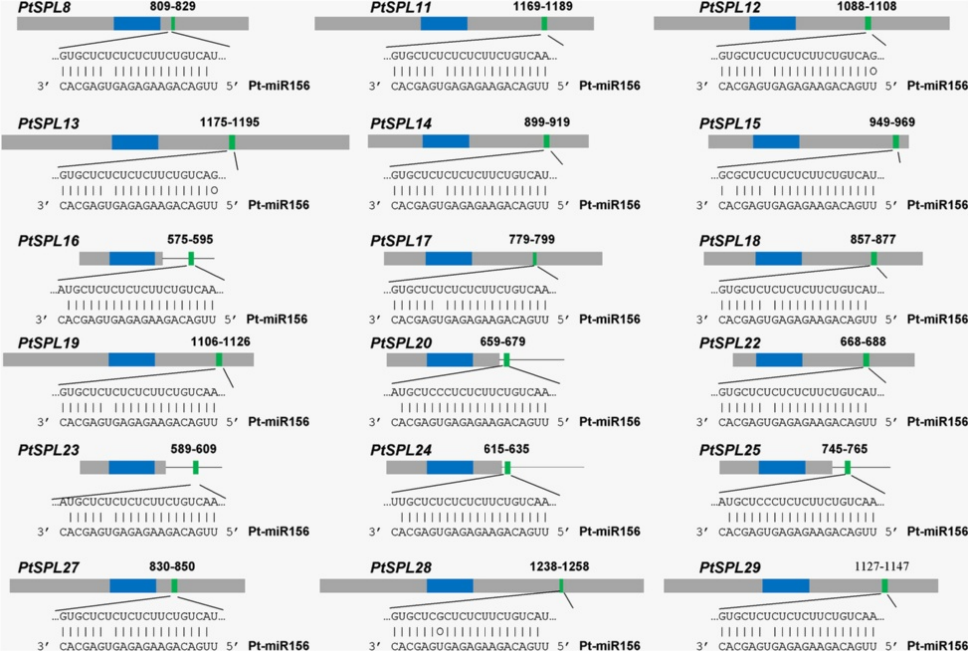
***PtSPLs *targeted by miR156.** Heavy grey lines represent ORFs. The lines flanking ORFs represent 3’-UTR. The blue lines represent SBP domain. miRNA complementary sites (green) with the nucleotide positions of *PtSPL* cDNAs are indicated. The RNA sequence of each complementary site from 5’ to 3’ and the predicted miRNA sequence from 3’ to 5’ are shown in the expanded regions.

It has been shown that 10 *AtSPLs* are regulated by miR156 [11]. The complementary sites of miR156 are in the coding regions or 3’ UTRs of *AtSPLs*. In order to know miR156-medicated posttranscriptional regulation of *PtSPLs*, we searched coding regions and 3’ UTRs of all *PtSPLs* for targets of *P. trichocarpa* miR156a–miR156j on the psRNATarget server using default parameters [45]. The results showed that 18 *PtSPLs* were potential targets of miR156 (Figures 9 and 10). MiR156-targeting sites in 13 *PtSPLs* belonging to G1 and G2 locate in the last exon and encode the conserved peptide ALSLLS. The target sites for other 5 *PtSPLs* belonging to G5 locate in the 3’ UTRs close to the stop codons (Figure 10). Consistently, *AtPSLs* clustering in G1, G2 and G5 are targets of miR156 in *Arabidopsis*. It suggests that miR156-mediated posttranscriptional regulation of SPLs is conserved in *P. trichocarpa* and *Arabidopsis*.

## Discussion

SPLs are plant-specific transcription factors containing a highly conserved SBP (SQUAMOSA PROMOTER BINDING PROTEIN) domain. It can specifically bind to the promoters of floral meristem identity gene *SQUAMOSA* and its orthologous genes and plays important regulatory roles in plant growth and development [46-49]. The genes encoding *SPLs* have been identified from various plant species, such as *Arabidopsis*[2,10,23,26], maize [30], *Antirrhinum majus*[3], rice [50], silver birch [51], and *S. miltiorrhiza*[41]. *SPL* genes exist as a large gene family in plants. The number of *SPLs* in *Arabidopsis*, rice, *P. patens*, maize and tomato is 16, 19, 13, 31 and 15, respectively [4-9]. Availability of the whole genome sequence allows us to perform genome-wide identification of *SPLs* in *P. trichocarpa*. Analysis of three versions of the annotated *P. trichocarpa* genome showed the existence of 28 full-length *PtSPLs*, which distribute on 14 chromosomes. It is the first attempt to analyze the *PtSPL* gene family. The results provide a basis for elucidating the functions of *SPLs* in *P. trichocarpa*, a model forest tree.

The number of *SPL* genes in *P. trichocarpa* is much greater than that in *Arabidopsis*, rice, *P. patens* and tomato, although it is similar to the number of maize *SPLs*[4-9]. Sequence homologous analysis suggests that gene duplication plays an important role in *SPL* gene expansion in *P. trichocarpa*. A total of 11 pairs of intrachromosome-duplicated *PtSPLs* were identified in this study. All of them clustered together in the phylogenetic tree (Figure 2). It is consistent with previous findings for generation and maintenance of gene families in other organisms, such as mouse, human and *Arabidopsis*[52,53]. Actually, gene duplication has been reported for many plant transcription factor gene families, such as *MYB*, *AP2*, *MADS* and so on [54-56] and duplicated *SPL* gene pairs have been identified in *Arabidopsis* (*AtSPL10/11*, *AtSPL4/5* and *AtSPL1/12*) and rice (*OsSPL2/19*, *OsSPL3/12*, *OsSPL4/11*, *OsSPL5/10* and *OsSPL16/18*) [57-61]. However, the number of homologous *PtSPL* gene pairs is obviously greater than that in *Arabidopsis* and rice, indicating that more segment duplication events happened in *Populus* and most *SPL* genes in *Arabidopsis* and *Populus* expanded in a species-specific manner [62-64].

Comparative analysis of *P. trichocarpa PtSPLs* and *Arabidopsis AtSPLs* revealed many conserved sequence features. For instance, all of the deduced proteins contain the highly conserved SBP domain with about 78 amino acid residues. The intron position and intron phase in the SBP-domain-encoding regions are also conserved among all *SPL* genes in *P. trichocarpa* and *Arabidopsis*, indicating that plant *SPL* genes originate from a common ancestor. Based on the neighbor-joining (NJ) phylogenetic tree constructed using MEGA 5.1., 44 SPL proteins from *P. trichocarpa* and *Arabidopsis* were found to cluster into 6 groups. Each group includes at least a PtSPL and one AtSPL. The intron number and intron phase are similar for PtSPLs and AtSPLs within a group. The results suggest the conservation between *P. trichocarpa PtSPLs* and *Arabidopsis AtSPLs*.

It has been shown that *AtSPLs* play significant regulatory roles in a variety of developmental processes in *Arabidopsis*. For instance, morphological traits of cauline leaves and flowers are regulated by *AtSPL2*, *AtSPL10* and *AtSPL11*[19]. Juvenile-to-adult growth phase transition and leaf initiation rate are controlled by the redundant action of *AtSPL9* and *AtSPL15*[21]. Pollen sac development, male fertility and GA biosynthesis and signaling are regulated by *AtSPL8*, a member of G3 [23-25]. Cu homeostasis in *Arabidopsis* is regulated by the member of group 6, *AtSPL7*[22]. In this study, we found that many motifs were unique to or mainly existed in a group of SPLs. It is consistent with the redundant roles of AtSPLs in a group and indicates that the members of PtSPLs in the same group may play similar roles as their *Arabidopsis* counterparts. The function of SPLs in different groups could be functionally distinct. On the other hand, three PtSPL-specific motifs, including motifs 11, 19 and 23, were identified, suggesting that some PtSPLs may play species-specific roles. Consistently, most of paralogous *PtSPL* gene pairs in the same group show similar expression patterns, whereas a few of them exhibit differential patterns. The results indicate subfunctionalisation and neofunctionalisation of SPLs within a plant species and among different species.

MiR156-medicated posttranscriptional regulation is important for the function of a subset of *SPLs*[11,41,65]. Target prediction showed that all *PtSPLs* in groups 1, 2 and 5 were regulated by miR156. The complementary sites of miR156 locate in the coding region of G1 and G2 *SPLs*, whereas it locates in 3’ UTR of G5 *SPLs*. It is consistent with the results from *Arabidopsis SPLs* and suggests the conservation of miR156-mediated posttranscriptional regulation in plants.

## Conclusion

In this study, a total of 28 full-length SPLs were identified from the whole genome sequence of *P. trichocarpa*. Through a comprehensive analysis of gene structures, phylogenetic relationships, chromosomal locations, conserved motifs, expression patterns and miR156-mediated posttranscriptional regulation, the *PtSPL* gene family was characterized and compared with *SPLs* in *Arabidopsis*. The results showed that 28 PtSPLs and 16 AtSPLs clustered into 6 groups. Many PtSPLs and AtSPLs within a group are highly conserved in sequence features, gene structures, motifs, expression patterns and posttranscriptional regulation, suggesting the conservation of plant SPLs within a group. However, significant differences were observed for *SPLs* among groups. In addition, various motifs were identified in PtSPLs but not in AtSPLs. It suggests the diversity of plant SPLs. The results provide useful information for elucidating the functions of *SPLs* in *P. trichocarpa*.

## Methods

### Identification of *PtSPL* genes

The nucleotide sequences and deduced amino acid sequences of 16 known *SPL* genes in *Arabidopsis*[2,4] were obtained from the TAIR database (http://www.arabidopsis.org) (Additional file 2). The SBP domain of *AtSPLs* was identified using Pfam (http://pfam.sanger.ac.uk). BLAST search of *PtSPLs* against *Populus trichocarpa* v1.1, v2.2 and v3.0 was carried out using *AtSPL* SBP as the query sequences [32] (http://genome.jgi-psf.org/Poptr1_1/Poptr1_1.home.html,http://www.phytozome.net/poplar.php#B). An e-value cut off of 1e^−5^ was applied to the recognition. We also searched the databases for SBP using the keywords search tool on the web servers. Protein sequences retrieved from *Populus trichocarpa* v1.1, v2.2 and v3.0 were then aligned and combined based on sequence identities.

### Chromosome location and sequence feature analyses

Chromosome locations of *PtSPL* genes were determined by BLAST analysis of *PtSPLs* against *Populus trichocarpa* v3.0 (http://www.phytozome.net/poplar.php#B). Paralogous gene pairs were analyzed on the Plant Genome Duplication Database (PGDD) server (http://chibba.agtec.uga.edu/duplication/index/locus) with display range for 100 kb. The approximate date of the duplication events was calculated using T = Ks/2λ by assuming clock-like rates (λ) in *Populus* for 9.0 × 10^−9^[32,57,66]. Synonymous substitutions (Ks) values of paralogous gene pairs were calculated using DnaSP [67]. The theoretical isoelectric point (p*I*) and molecular weight (Mw) were predicted using the Compute pI/Mw tool on the ExPASy server (http://web.expasy.org/compute_pi/) [68]. The intron/exon structure of *SPL* genes was predicted with the Gene Structure Display Server (http://gsds.cbi.pku.edu.cn/chinese.php) [69].

### Phylogenetic construction and motif analysis

Phylogenetic trees were constructed using the neighbor-joining (NJ) method in MEGA5.1. Branching reliability was assessed by the bootstrap re-sampling method using 1,000 bootstrap replicates. Only nodes supported by bootstrap values greater than 50% were analyzed. Conserved domains of PtSPLs were identified using Pfam (http://pfam.sanger.ac.uk) and by BLAST analysis of protein sequences against the Conserved Domain Database (CDD, http://www.ncbi.nlm.nih.gov/Structure/cdd/wrpsb.cgi) with the expected e-value threshold of 1.0 and the maximum size of hits to be 500 amino acids [70]. The 78 amino acids of SBP domain were aligned using clustalW. Sequence logos were generated using the weblogo platform (http://weblogo.berkeley.edu/). Potential protein motifs were predicted using the MEME package (http://meme.sdsc.edu/meme/) with the following parameters applied. It includes the distribution of motifs: zero and one per sequence, maximum number of motifs to find: 25, minimum width of motif: 8, and maximum width of motif: 150. An e-value cut off of 1e^−10^ was applied to the recognition.

### Quantitative real-time reverse transcription-PCR (qRT-PCR)

*P. trichocarpa* plants were grown in an artificial climate chamber for about one year. Young leaves (2nd–3rd from the top), mature leaves (12th from the top), young stems (1st–3rd from the top), young roots, tissues of developing secondary xylem and phloem from the 4th–6th and 12th–25th internodes from the top of *P. trichocarpa* plants were collected. Three biological repeats were carried out. Total RNA was extracted using the plant total RNA extraction kit (Aidlab, China). Genomic DNA contamination was eliminated by pre-treating total RNA with RNase-free DNase (Promega, USA). RNA integrity was analyzed on a 1.2% agarose gel and its quantity was determined using a NanoDrop 2000C Spectrophotometer (Thermo Scientific, USA). Total RNA was reverse-transcribed by Superscript III Reverse Transcriptase (Invitrogen, USA). qRT-PCRs were carried out in triplicate for each tissue sample using gene-specific primers (Additional file 6) as described previously [71]. The program used for qRT-PCR is as follows: predenaturation at 95°C for 30s, 40 cycles of amplification at 95°C for 5 s, 60°C for 18 s and 72°C for 15 s. The length of amplicons was between 80 bp and 250 bp. *Actin* was used as a reference gene as described previously [72]. Dissociation curve was used to assess amplification specificity. Relative abundance of transcripts was analyzed using the comparative Ct method [73]. The arithmetic formula, 2-ΔΔCq, was used to achieve results for relative quantification. Cq represents the threshold cycle. Standardization of gene expression data from three biological replicates was performed as described [74]. For statistical analysis, ANOVA (analysis of variance) was calculated using SPSS (Version 19.0, IBM, USA). P < 0.05 was considered statistically significant.

### Microarray data analysis

Microarray data of *PtSPLs* was obtained by the ePlant-tissue expression tool at PopGenIE (http://www.popgenie.org/). The data was gene-wise normalized and then analyzed using the average linkage clustering technique in Cluster 3.0 [75].

### Prediction of *PtSPLs* targeted by miR156

The sequences of *P. trichocarpa* miR156a–miR156j were obtained from miRBase [36] (http://www.mirbase.org/). *PtSPLs* targeted by miR156 were predicted by searching the coding regions and 3’ UTRs of all *PtSPLs* for complementary sequences of *P. trichocarpa* miR156a–miR156j on the psRNATarget server using default parameters [45] (http://plantgrn.noble.org/psRNATarget/?function=3).

### Availability of supporting data

The data sets supporting the results of this article are included within the article and its additional files.

## Abbreviations

CuRE: Cu-response element; Mw: The molecular weight; NJ: Neighbor-joining; NLS: Nuclear location signal; pI: Isoelectric point; qRT-PCR: Quantitative realtime reverse transcription-PCR; SBP: SQUAMOSA PROMOTER BINDING PROTEIN; Zn1: Zinc finger 1; Zn2: Zinc finger 1.

## Competing interests

The authors declare that they have no competing interests.

## Authors’ contributions

CL contributed to bioinformatics and qRT-PCR analyses and participated in writing the manuscript. SL designed the experiment, performed bioinformatics analysis and wrote the manuscript. Both authors have read and approved the version of manuscript.

## Supplementary Material

Additional file 1 **Sequence features of PtSPLs in *P. trichocarpa. *** **Protein length, intron number, p** ***I *** **and molecular weight of SPLs in *P. trichocarpa *** **are shown.** Click here for file

Additional file 2 **Sequence features of AtSPLs in *A. thaliana.*** Gene IDs, protein length, intron number, p*I* and molecular weight of SPLs in *A. thaliana* are shown. Click here for file

Additional file 3 **Neighbor-joining (NJ) phylogenetic tree constructed for 77 SPLs from *P. trichocarpa, Arabidopsis, *** **rice and *S. miltiorrhiza.*** The groups of homologous genes identified and bootstrap values are shown. The reliability of branching was assessed by the bootstrap re-sampling method using 1,000 bootstrap replicates. Bootstrap values are shown below nodes. Click here for file

Additional file 4 **Intron distribution on SBP domains of *Populus *** **and *Arabidopsis.*** Intron distribution on SBP domains of *Populus* and *Arabidopsis* are shown. Click here for file

Additional file 5 **Alignment of the ANK/ANK-2 domain.** The ANK/ANK-2 domain is indicated by solid lines. Click here for file

Additional file 6 **Primers used for qRT-PCR analysis of *PtSPL *** **genes.** Complete set of primers used for qRT-PCR. Click here for file
