# Supplementary material for: Molecular characterization of the SPL gene family in Populus trichocarpa
Source: BMC Plant Biol. 2014 May 15;14:131. doi: 10.1186/1471-2229-14-131 (PMC4035897; doi:10.1186/1471-2229-14-131)
Supplement: Additional file 3 — Neighbor-joining (NJ) phylogenetic tree constructed for 77 SPLs from P. trichocarpa, Arabidopsis, rice and S. miltiorrhiza. The groups of homologous genes identified and bootstrap values are shown. The reliability of branching was assessed by the bootstrap re-sampling method using 1,000 bootstrap replicates. Bootstrap values are shown below nodes. [file 1471-2229-14-131-S3.doc]

**Additional file 3. Neighbor-joining (NJ) phylogenetic tree constructed for 77 SPLs from *P. trichocarpa*, *Arabidopsis*, rice and *S. miltiorrhiza.*** The groups of homologous genes identified and bootstrap values are shown. The reliability of branching was assessed by the bootstrap re-sampling method using 1,000 bootstrap replicates. Bootstrap values are shown below nodes.
