# Supplementary material for: Molecular characterization of the SPL gene family in Populus trichocarpa
Source: BMC Plant Biol. 2014 May 15;14:131. doi: 10.1186/1471-2229-14-131 (PMC4035897; doi:10.1186/1471-2229-14-131)
Supplement: Additional file 5 — Alignment of the ANK/ANK-2 domain. The ANK/ANK-2 domain is indicated by solid lines. [file 1471-2229-14-131-S5.doc]

**Additional file 5. Alignment of the ANK/ANK-2 domain.** The ANK/ANK-2 domain is indicated by solid lines.
